# Comparative prototypes and metabolites of Du-zhi pill in normal and cerebral ischemia rats by UHPLC-Q-TOF-MS/MS method

**DOI:** 10.1016/j.heliyon.2024.e25059

**Published:** 2024-01-20

**Authors:** Huaqing Lai, Guanghuan Tian, Fuzhu Pan, Jianyong Zhang, Hongwei Wu

**Affiliations:** a School of Pharmacy, Zunyi Medical University, Zunyi, 563000, China; b Institute of Chinese Materia Medica, China Academy of Chinese Medical Sciences, Beijing, 100700, China; c Science and Technology Innovation Center, Guangzhou University of Chinese Medicine, Guangzhou, 510405, China

**Keywords:** Du-zhi pill, UHPLC-Q-TOF-MS/MS, Metabolite profile, Metabolic pathways

## Abstract

Du-Zhi pill (DZP) is widely used as a Chinese medicine in treating cerebral ischemia. UHPLC-Q-TOF-MS/MS techniques were used to detect and identify the metabolites in rat brain samples of normal and middle cerebral artery occlusion (MCAO) model rats administered with DZP. It was tentatively found that 43 prototypes and 93 metabolites could be identified in rat brain samples. Normal and MCAO model rat brain samples contained 19 prototype components. Eight prototype components were only detected in normal rat brain samples, while 16 were found only in MCAO model rat brain samples. It was determined that 47 metabolites had been identified in the normal rats, while 86 had been placed in MCAO model rats. There were 40 common metabolites in both normal and MCAO model rat brain samples. Seven metabolites were only detected in normal rat brain samples, while 46 were found only in MCAO rat brain samples. The comparison of metabolites in brain samples of normal and MCAO rats showed apparent differences. It was discovered that glucuronidation, methylation, acetylation, and sulfation are phase II metabolic routes of DZP, while hydrogenation, hydroxylation, and dehydroxylation are phase I metabolic routes. Moreover, hydrogenation, glucuronidation, hydroxylation, and methylation were the main metabolic pathways because of the number of metabolites identified in these metabolic pathways. The results provide a valuable reference for further research into effective substances of DZP for treating cerebral ischemia.

## 1 Introduction

Stroke is a significant health challenge worldwide because it is a leading cause of death and disability among adults [[1], [2], [3]]. Du-Zhi pill (DZP), a Chinese patented medicine, consists of 12 herbal medicines, including Rehmanniae Radix (*Rehmannia glutinosa* Libosch.), Morindae officinalis Radix (*Morinda officinalis* How), Astragali Radix [*Astragalus membranaceus* (Fisch.) Bunge], Hirudo (*Whitmania pigra* Whitman), Paeoniae Radix Rubra (*Paeonia lactiflora* Pall.), Epimedii Folium (*Epimedium brevicornu* Maxim.), Leonuri Herba (*Leonurus japonicus* Houtt.), Cynanchi Atrati Radix Et Rhizoma (*Cynanchum atratum* Bge.), Eucommiae Cortex (*Eucommia ulmoides* Oliv.), Acori tatarinowii Rhizoma (*Acorus tatarinowii* Schott), Angelicae Sinensis Radix [*Angelica sinensis*(Oliv.)Diels], Lycopodii Herba (*Lycopodium japonicum Thunb*.). As a traditional Chinese medicine prescription used to treat cerebral vessel disease, DZP is widely known for its effectiveness. Hence, it is widely used by Chinese patients suffering from cerebral ischemia. The latest studies suggest that DZP has a significant clinical therapeutic effect on ischemic stroke [4]. In particular, DZP can significantly decrease the infarction size and neurological deficit scores. Moreover, DZP can reduce neuroinflammation and inhibit related metabolic pathways [5]. In our previous study, a total of 176 DZP components were identified, including 50 flavonoids, 31 phenolic acids, 25 terpenoids, five alkaloids, and 65 other components [6]. It was generally known that the chemical components that can enter the brain through the blood-brain barrier can play a role in the treatment of CNS diseases. Therefore, it is essential to study DZP metabolism to gain a comprehensive understanding of the in *vivo* processes of DZP. We have recently conducted a study on DZP metabolism *in vivo*, mainly focusing on identifying their metabolites in plasma, urine, and feces [6]. However, the components of DZP, which can enter the brain to have direct treatments for cerebral ischemia, have not been studied.

Many metabolic studies in the past have been performed on normal animals [[7], [8], [9]]. However, pathological and normal organisms have different drug metabolism characteristics [10,11]. The destruction of the blood-brain barrier under cerebral ischemia has a significant influence on the translation and metabolism of the drugs in the brain. Many studies have shown that drug compounds have different metabolism characteristics, such as tissue distribution and pharmacokinetics, in normal and pathological conditions [[12], [13], [14]].

It is necessary to compare the metabolism of DZP in normal and cerebral ischemia model rats in order to gain a better understanding of DZP metabolism in these rats. In recent years, high-resolution mass spectrometry has become a powerful and reliable analytical technique for metabolite identification due to its excellent accurate mass measurement, sensitivity, and high resolution [[15], [16], [17]]. In this study, ultra-high performance liquid chromatography/quadrupole time-of-flight mass spectrometry (UHPLC-Q-TOF-MS) was used to investigate the metabolic profile of DZP in normal and middle cerebral artery occlusion (MCAO) model rat brain samples and compare the differences between normal and cerebral ischemia pathological state. Based on the metabolism study, MCAO model rat brain samples may provide a valuable reference for further research into effective substances for treating cerebral ischemia. Further, an integrated strategy for identifying TCM prescription metabolites was proposed.

## 2 Materials and methods

### 2.1 Chemicals and reagents

There were 12 reference compounds purchased from Chengdu Desite Biotechnology Co., Ltd. (Chengdu, China), including nystose, gallic acid, 6-hydroxypurine, 2,6-dihydroxypurine, catalpol, calycosin-7-O-β-D-glucoside, epimedin A, imperatorin, pinoresinol diglucoside, senkyunolide I, lycoclavanol, astragaloside A (purity>98 %, all). UHPLC analysis was performed using mass spectrometry grade acetonitrile, methanol, and formic acid from Thermo Fisher Scientific Co., Ltd.

Jilin Aodong Pharmaceutical Group Co. Ltd. provided the Du-zhi pill powder (the mixed powder before pill making). The purified water for preparing the mobile phase was supplied by Hangzhou Wahaha Group Co., Ltd. All chemicals used throughout this study were of HPLC grade unless stated otherwise.

### 2.2 UHPLC-Q-TOF-MS/MS conditions

UHPLC-Q-TOF-MS/MS analysis was performed on a triple TOF 5600 MS/MS system (AB SCIEX, CA, USA) coupled with a Shimadzu UHPLC system (Kyoto, Japan). According to the final chromatographic separation conditions, the following parameters were optimal: the mobile phase was 0.1 % formic acid/water (A) and acetonitrile (B); the chromatographic column was ACQUITY BEH C18 columns (2.1 × 100 mm, 1.7 μm; Waters, USA); the injection volume was 10 μL; the flow rate was 0.2 mL/min, and the temperature column was 30 °C. Gradient elution programs were optimized as follows: 0–8 min, 5–20 % B; 8–22 min, 20–45 % B; 22–28 min, 45–100 % B; 28–32 min, 100–100 % B; 32–33 min, 100–5% B; 33–37 min, 5–5% B.

The detection system used was a Triple TOF 5600 with Dual-Spray ion sources. Parameter settings were as follows: turbo spray temperature: 550 °C; ion spray voltage: 5.5 kV in the positive ion mode and −4.5 kV in negative ion mode; nebulizer gas (gas 1): 50 psi; heater gas (gas 2): 50 psi; curtain gas: 25psi; collision energy (CE): 35 eV; collision energy spread (CES): 15 eV and declustering potential (DP): 60V. In the full scan, the mass range was *m/z* 50–1500 Da, and the accumulation time was 200 ms. Both positive and negative ion modes were used in the instrument.

### 2.3 Animal experiments

Sipeifu (Beijing) Biotechnology Co. Ltd. (Beijing, China) provided adult male Sprague Dawley rats (certificate no. SCXK (Jing) 2019-0010) weighing 230 ± 10 g. There were controlled environmental conditions for all rats (12 h light/dark cycle; relative humidity, 50 %; temperature, 22 °C). Water and food were freely available to all rats. All experimental procedures were approved by the China Academy of Chinese Medical Science's Administrative Panel on Laboratory Animal Care (Ethical Approval No. ERCCACMS21-2106-17). Institutional guidelines and ethics were followed in all animal experiments.

By a previous method, MCAO was performed using the intraluminal filaments method [18]. The MCA origin was occluded by inserting a monofilament nylon suture with a round tip from the left external carotid artery into the internal carotid artery lumen.

Six rats in the Sham group and MCAO group were randomly selected to verify the MCAO model. The Longa 5 neurological deficient scores were performed to evaluate neurologic impairment [19]. The brain sections 2 mm thick were stained with 2,3,5-triphenyl tetrazolium chloride at a concentration of 2 % (TTC, w/v, Sigma) at 37 °C for 0.5 h.

After the successful establishment of the evaluation animal MCAO model, Six MCAO rats were randomly divided into two groups with three rats per group (Group I, the dosed MCAO group; Group II, the blank MCAO group). Six rats were randomly divided into two groups with three rats per group (Group I, the dosed group; Group II, the blank group). Powders of the DZP sample (87.15 mg/mL) were dissolved in a water solution and ultrasonically vibrated for 10 min. A 12-h fast with free access to water was performed before the experiment on all rats. In all experimental groups, rats were administered the drug by oral gavage for one day at a dose equivalent to clinical doses of 1.78 g/kg [6]. Equivalent volumes of 0.9 % physiological saline were administered to the blank groups as a control.

The drugs were administered orally at 8:00 a.m. After 2 h after the oral administration, the rats were sacrificed, followed by brain removal. Before analysis, all biological samples obtained were frozen at −80 °C.

### 2.4 Collection and preparation of biological samples

After crushing and weighing the brain samples (1.0 g), they were extracted using methanol (4 mL) by ultrasonic treatment for 30 min. For the next step, centrifuge at 4 °C for 10 min at 13400 *g*. Under a stream of nitrogen at 30 °C, the supernatant was evaporated to dryness in another tube. It was then reconstituted with 100 μL of methanol and centrifuged at 4 °C at 13400 *g* for 10 min. All samples were filtered through a 0.22 μm pore membrane to analyze sample solutions and injected into the UHPLC-Q-TOF-MS system.

### 2.5 Data processing procedures

**Fig. 1:**
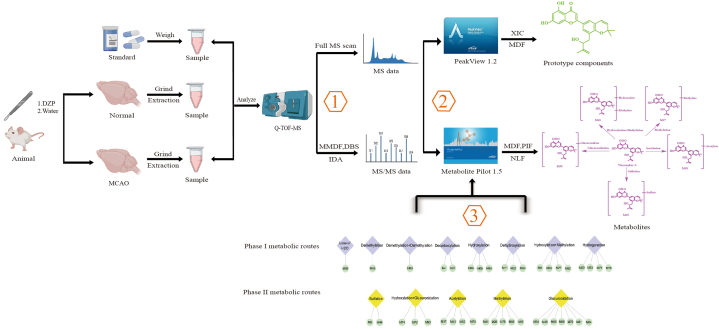
Research route for systematic analysis of DZP *in vivo*.

Fig. 1 illustrates how raw data were processed to systematically identify multiple constituents of DZP metabolites following oral administration to rats.

All data were processed on Metabolite Pilot 1.5, and the data processing parameters were set as follows: mass range, 50–1500 Da; retention time tolerance was 0.2 min; positive adducts (+H, +Na, +NH_4_, +K), negative adducts (–H, +COOH, +Cl); retention time range, 0.1–37.0 min; mass accuracy tolerance at ± 10 ppm. The parameters of MDF were mass window 0.04 Da and defect padding 100 mDa.

As a first step, online data acquisition, including a full mass scan, was performed, and accurate MS/MS data sets were obtained using dynamic background subtraction (DBS) and multiple mass defect filter (MMDF)-dependent methods. With DBS, background and matrix-related MS/MS ions will be eliminated, and drug-related MS/MS ions will be more easily identified. It is possible to exploit this advantage to capture low levels of metabolites.

Additionally, various data-mining tools were used to process the acquired data post-acquisition. The potential metabolites of DZP in healthy and model rats were searched using various data analysis tools containing product ion filter (PIF), mass defect filter (MDF), and neutral loss filter (NLF) in Metabolite Pilot 1.5. Molecular weights and elemental compositions of possible metabolites were predicted using the MDF and XIC.

Based on accurate mass measurements, relevant biotransformation information, fragmentation patterns of the parent drug, and MS/MS spectra of metabolites have been reported; the third step consisted of identifying the chemical structure of metabolites. Metabolites were analyzed using Metabolite Pilot 1.5 software by comparing samples and controls to detect possible bio-transformations, including hydrolysis, decarboxylation, hydrogenation, hydroxylation, dehydroxylation, demethylation, acetylation, methylation, glucuronidation, and sulfation. Structures were characterized based on the retention times of the reference compounds and fragmentation rules in the literature.

## 3 Results and discussion

### 3.1 Evaluation of the MCAO rats

**Fig. 2:**
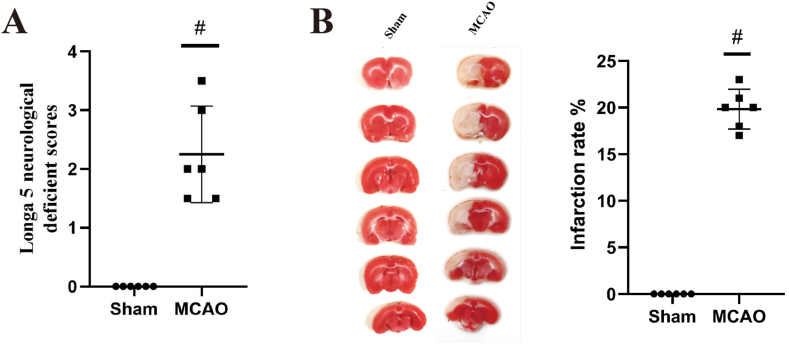
Evaluation of the MCAO Rats. A Longa 5 neurological deficit score (*n* = 6). B TTC staining and the calculated infarction rate (*n* = 6). The data are expressed as mean ± SD. ^#^*p* < 0.05 compared to the sham group.

As shown in Fig. 2A-B, rats in the MCAO group demonstrated an obvious increase in infarction rate and neurological deficit scores when compared with the sham group, indicating obvious cerebral injury after MCAO (*P* < 0.05). Then, the metabolism study of DZP after oral administration, based on the MCAO model, was conducted.

### 3.2 Identification of prototype components in rats

The prototype components derived from DZP were identified based on the mass data by comparing them with the compounds detected in the DZP extract in our previous study [6]. The metabolites of the prototype components were further identified in the brain samples after oral administration but did not exist in the DZP extract.

**Fig. 3:**
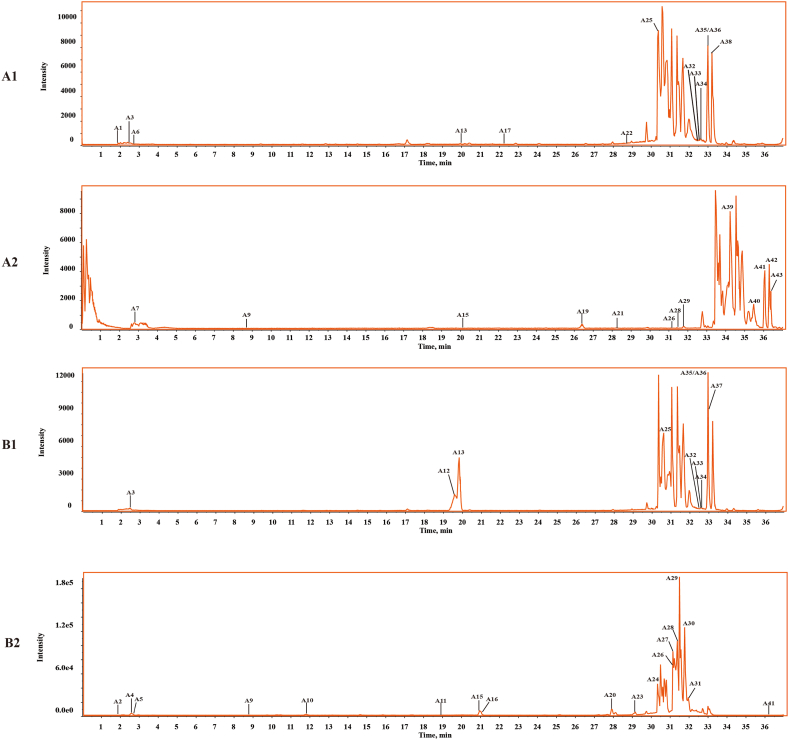
The base peak intensity chromatograms of DZP prototype components in normal and MCAO model rat brain samples: normal (A1) and MCAO model (B1) in the negative ion mode; normal (A2) and MCAO model (B2) in positive ion mode.

Based on the identified compounds in the DZP extract [6], **s**creening prototypic compounds in the brain samples was conducted using the XIC in peakview 1.2 software. Further confirmation of the structures was achieved through MS/MS experiments. Fig. 3 shows the BPI chromatograms for normal (Fig. 3 A1, Fig. 3 A2) and MCAO model rat brains (Fig. 3 B1, Fig. 3 B2). A total of 43 prototype components were detected in *vivo* in both the MCAO group treated by DZP (MCAO group) and the normal group treated by DZP (normal group). Among the 43 prototype components, ten flavonoids, six coumarins, 5 terpenoids, and 22 others were found (Table 1). Flavonoids, coumarins, and terpenoids were the main components that were exposed in *vivo*.

**Table 1:** Identification of prototype components in rats of DZP by UHPLC-Q-TOF-MS/MS.

Peak No	t_R_ (min)	Observed *m/z*	Mass Error (ppm)	Adducts	Formula	Fragment (±)	Identification	Source	Distribution
A1	1.97	168.0436	4.6	M^-^	C_8_H_8_O_4_	146.0591,121.0195,65.9714	Vanillic Acid [6]	DZ, DG, CS, DH, SCP	N
A2	1.98	689.2060	−7.4	[M+Na]^+^	C_24_H_42_O_21_	568.1788,537.1889,485.0448,383.9972	Nystose^a^	BJT	M
A3	2.45	204.9885	−6.5	[M+Cl]^-^	C_7_H_6_O_5_	171.0569,154.9870,125.5589,79.6623	Gallic Acid^a^	DZ, CS	N, M
A4	2.50	423.1407	−1.8	[M+Na]^+^	C_22_H_24_O_7_	307.0392,289.0652,137.0416	Brevicornin [6]	YYH	M
A5	2.59	137.0447	−8.0	[M+H]^+^	C_5_H_4_N_4_O	119.0320,94.0376,58.0286	6-Hydroxypurine^a^	SZ	M
A6	2.72	440.1287	−8.3	[M − H]^-^	C_19_H_19_N_7_O_6_	346.0762,267.0885,233.9773,189.1931	Folic Acid [6]	DG	N
A7	2.80	175.0220	−3.8	[M+Na]^+^	C_5_H_4_N_4_O_2_	154.9728,129.0301,112.9845	2,6-Dihydroxy-purine^a^	SZ	N
A8	3.70	385.1125	5.2	[M+Na]^+^	C_15_H_22_O_10_	357.0936,301.5898,242.0956,219.5987	Catalpol^a^	DZ, DH	M
A9	8.86	447.1323	8.2	[M+H]^+^	C_22_H_22_O_10_	401.2205,301.1351,283.1640	Calycosin-7-O-β-D-Glucoside^a^	HQ	N, M
A10	13.56	376.1791	9.4	[M + NH_4_]^+^	C_20_H_22_O_6_	337.1932,301.1364,271.2565,177.0879	Pinoresinol [6]	DZ	M
A11	18.91	838.2851	−4.7	M^+^	C_39_H_50_O_20_	703.5714,675.1118,543.4896,301.1363	Epimedin A^a^	YYH	M
A12	19.37	383.1011	10.0	[M + HCOO]^+^	C_16_H_18_O_8_	313.1231,225.1421,197.1365	3-O-P-Coumaroy-lquinic Acid [6]	YYH	M
A13	19.91	445.07764	0.6	[M − H]^-^	C_21_H_18_O_11_	269.0440,232.3238,175.0237,113.0232,85.0292	Baicalin [6]	HQ, DG, CS	N, M
A14	20.12	339.0844	1.3	[M+Na]^+^	C_17_H_16_O_6_	299.1248,271.1017,249.1164,163.1332	Byakangelicol [6]	DG	M
A15	20.13	271.0983	6.6	[M+H]^+^	C_16_H_14_O_4_	249.1164,225.1340,157.0554	Imperatorin^a^	DG	N, M
A16	20.56	700.2856	6.3	[M + NH_4_]^+^	C_32_H_42_O_16_	622.6784,519.2307,478.2148,299.1241	Pinoresinol Diglucoside^a^	DZ	M
A17	22.31	447.0942	2.0	[M − H]^-^	C_21_H_20_O_11_	401.3273,360.2741, 301.2592,280.9994,227.1943	Quercitrin [6]	YYH	N
A18	22.62	469.1061	−9.3	[M+Na]^+^	C_22_H_22_O_10_	429.1150,237.0715,175.0900	Sissotrin [6]	HQ	M
A19	26.56	263.0661	−7.1	[M+K]^+^	C_12_H_16_O_4_	225.1692,158.0921	Senkyunolide I^a^	DG	N, M
A20	27.65	398.2365	9.7	[M + NH_4_]^+^	C_24_H_28_O_4_	376.2549,300.1355	Angelicide [6]	DG	N, M
A21	28.21	363.3245	3.0	[M+Na]^+^	C_22_H_44_O_2_	339.2947,301.1361,274.2676	Behenic Acid [6]	YYH	N, M
A22	28.72	269.0456	−1.2	[M − H]^-^	C_15_H_10_O_5_	241.0445,223.0344,195.0402,169.0616,136.9850	Baicalein [20]	DZ, CS	N
A23	29.14	438.1916	1.2	[M + NH_4_]^+^	C_25_H_24_O_6_	415.2061,351.3274,282.2000	Yinyanghuo A [6]	YYH	M
A24	29.90	337.0691	2.6	[M+Na]^+^	C_17_H_14_O_6_	283.0482,265.0075	Odoratin [6]	HQ	M
A25	30.65	503.3777	9.2	[M + HCOO]^+^	C_30_H_50_O_3_	481.9050,306.1021,171.1347,153.0970	Lycoclavanol^a^	SJC	N, M
A26	31.27	409.1289	7.5	[M+Na]^+^	C_21_H_22_O_7_	341.2634,242.2314,148.0968	Pteryxin [6]	DG	N, M
A27	31.29	493.3062	−3.4	[M+K]^+^	C_30_H_46_O_3_	426.3527,400.3366,341.2632,279.2307	3-Oxo-Olean-12-En-28-Oic Acid [6]	DH	M
A28	31.31	418.1624	0.4	M^+^	C_22_H_26_O_8_	376.9508,292.9717,131.1704	Syringaresinol [6]	DZ	N, M
A29	31.35	137.1328	2.1	[M+H]^+^	C_10_H_16_	81.0260,67.0806	γ-Terpinene [6]	CS	N, M
A30	31.90	785.4625	−7.2	[M+H]^+^	C_41_H_68_O_14_	703.5694,522.3512,438.3686	Astragaloside A^a^	HQ	M
A31	31.95	457.3631	−9.9	[M+H]^+^	C_30_H_48_O_3_	428.3683,369.2959,293.2628	Oleanic Acid [6]	YYH, CS, DH	M
A32	32.53	325.2372	−0.5	[M + HCOO]^+^	C_18_H_32_O_2_	281.2473,261.0044,223.0452	Linoleic Acid [6]	BJT,DG	N, M
A33	32.56	223.0418	7.9	[M − H]^-^	C_14_H_8_O_3_	207.1821,169.1055,74.9885	2-Hydroxyanthraqui-none [6]	BJT	N, M
A34	32.65	301.2365	−2.9	[M + HCOO]^+^	C_16_H_32_O_2_	283.5197,257.4956,229.3585,158.2161	N-Hexadecanoic Acid [6]	BJT, DG, SZ, CS	N, M
A35	33.01	327.2521	−2.7	[M + HCOO]^+^	C_18_H_34_O_2_	281.5554,255.4765,229.4167,175.2837	Oleic Acid [6]	YYH, BJT, CS	N, M
A36	33.02	283.2623	−6.9	[M − H]^-^	C_18_H_36_O_2_	263.4137,161.2475,135.1936	Octadecanoic Acid [6]	BJT, YYH	N, M
A37	33.04	327.1991	7.8	[M − H]^-^	C_21_H_28_O_3_	303.2529,271.2428,268.2338	Pyrethrin I [6]	CS	M
A38	33.26	303.2075	−3.3	[M+Cl]^+^	C_17_H_32_O_2_	285.5401,259.5155,201.3799	Methyl-11-Hexadecenoate [6]	SZ	N
A39	34.40	388.2084	−1.9	M^+^	C_19_H_32_O_8_	355.0413,167.1184,153.0817	Rehmaionoside C [6]	DH	N
A40	35.89	277.2128	−3.6	[M+Na]^+^	C_16_H_30_O_2_	253.9785,220.1694,158.9179	1,2–15,16-Diepoxyhe-xadecane [6]	DG	N
A41	36.30	403.1402	3.7	[M+H]^+^	C_21_H_22_O_8_	382.2856,312.2248,260.2402	Cartilaginomargina-din [6]	DG	N, M
A42	36.22	302.1391	1.4	[M + NH_4_]^+^	C_17_H_16_O_4_	183.0649	Pabulenol [6]	DG	N, M
A43	36.58	361.1615	−8.5	[M+H]^+^	C_20_H_24_O_6_	299.1361,219.1707	Lariciresinol [6]	DZ, DH	N, M

Note: t_R_: retention time, DZ:Eucommiae Cortex, BJT:Morinda officinalis, YYH:Epimedium brevicornu, HQ:Astragalus membranaceus, DG:Angelica sinensis, SZ:Hirudo, CS:Paeoniae Radix Rubra, YMC:Leonurus japonicus, DH:Rehmannia glutinosa, BW:Cynanchum Atratum, SCP:Acorus tatarinowii,SJC: Lycopodium japonicum.

N and M represented normal and model, respectively.

a Compound with a reference standard.

By comparing the two groups, the MCAO model and normal rats had different kinds and numbers of prototype components in brain samples. MCAO model rat brains contained 35 prototype components, while normal rat brains contained 27. Table 1 compares prototype components in brain samples from normal and MCAO model rats. Normal and MCAO model rat brain samples contained 19 common prototype components (A3, A9, A13, A15, A19, A20, A21, A25, A26, A28, A29, A32—A36, and A41—A43). It was discovered that A1, A6, A7, A17, A22, and A38—A40 could be found only in normal rat brain samples, while A2, A4, A5, A8, A10—A12, A14, A16, A18, A23, A24, A27, A30, A31, and A37 can only be detected in MCAO model rat brain samples. A marked difference was observed in the comparison of prototype components in brain samples from normal and MCAO rats. It is possible that the destruction of the blood-brain barrier following MCAO led to the different prototype components entering the brain. Moreover, the identification of prototype constituents can also contribute to the discovery and identification of the metabolites of DZP.

### 3.3 Identification of metabolites in rats

**Fig. 4:**
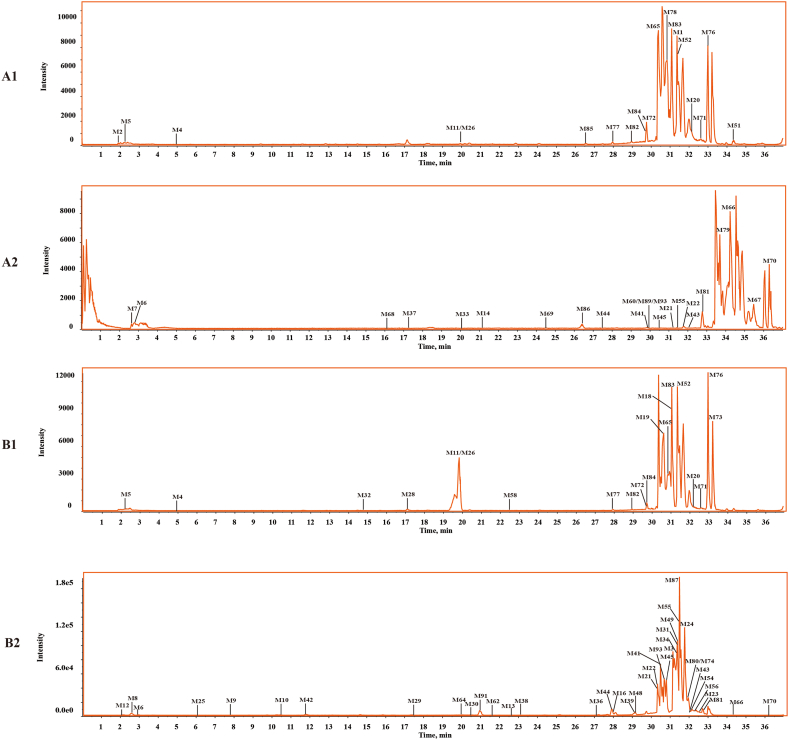
The base peak intensity chromatograms of DZP metabolites in normal and MCAO model rat brain samples: normal (A1) and MCAO model (B1) in the negative ion mode; normal (A2) and MCAO model (B2) in positive ion mode.

Based on the identification of the chemical components of DZP extract, various data-mining tools, including XIC, MDF, PIF, and NLF, were combined to identify DZP metabolites in rats. Fig. 4 shows the BPI chromatograms for normal (Fig. 4 A1, Fig. 4 A2) and MCAO model (Fig. 4 B1, Fig. 4 B2) rat brain samples. As a result, a total of 93 metabolites were detected and identified in the brain samples of the DZP-treated groups (normal and MCAO groups). In the brain samples of DZP-treated normal groups, 47 metabolite components were placed, and 86 metabolites were identified in the DZP-treated MCAO groups. All the metabolites have been listed in Table 2, and the potential metabolism pathways for the main types of constituents, such as flavonoids, coumarins, and terpenoids, are presented in Fig. 9.

**Table 2:** Identification of metabolites in rats of DZP by UHPLC-Q-TOF-MS/MS.

Peak No	t_R_ (min)	Observed*m/z*	Mass Error (ppm)	Adducts	Formula	Fragment (±)	Metabolite description	Distrib-ution
M1	31.42	149.0250	3.7	[M − H]^-^	C_8_H_6_O_3_	124.9213,115.5286,78.3732	Vanillic Acid + Loss of H_2_O	N
M2	1.96	359.0627	2.1	[M − H]^-^	C_14_H_16_O_11_	318.0982,267.0920,183.0279,168.0436	Vanillic Acid + Hydroxylation +Glucuronidation	N
M3	31.21	505.1803	7.9	[M+H]^+^	C_18_H_32_O_16_	400.3372,341.2637,239.2312	Nystose + Deglucose	M
M4	4.97	124.0159	−5.8	[M − H]^-^	C_6_H_5_O_3_	95.1373,78.9714	Gallic Acid + Decarboxylation	N, M
M5	2.38	248.9721	4.3	[M − H]^-^	C_7_H_6_O_8_S	204.9874,169.0201,113.0007	Gallic Acid + Sulfation	N, M
M6	2.94	201.0384	−5.0	[M+H]^+^	C_8_H_8_O_6_	174.0386,135.0298,78.9603	Gallic Acid +Hydroxylation + Methylation	N, M
M7	2.66	347.0627	5.3	[M+H]^+^	C_13_H_14_O_11_	318.0683,267.0675,171.0298	Gallic Acid + Glucuronidation	N
M8	2.81	347.1311	−7.3	[M+H]^+^	C_15_H_22_O_9_	255.1297,219.5524,160.0711	Catalpol + Dehydroxylation	M
M9	7.84	393.1382	−2.5	[M+H]^+^	C_16_H_24_O_11_	376.0981,357.6841,301.1197	Catalpol +Hydroxylation + Methylation	M
M10	10.70	377.1453	2.8	[M+H]^+^	C_16_H_24_O_10_	357.1352,301.1369,219.1721	Catalpol + Methylation	M
M11	19.79	429.1171	−4.6	[M − H]^-^	C_22_H_22_O_9_	301.1215,246.1141,225.1415	Calycosin-7-O-β-D-Glucoside + Dehydroxylation	N, M
M12	2.03	433.1151	5.1	[M+H]^+^	C_21_H_20_O_10_	422.1343,301.0387,291.0642,246.5130, 191.0345	Calycosin-7-O-β-D-Glucoside + Demethylation	M
M13	22.62	429.1153	−6.3	[M+H]^+^	C_22_H_20_O_9_	343.2788,301.1359,246.0766,158.0910	Calycosin-7-O-β-D-Glucoside +Loss of H_2_O	M
M14	21.31	639.1609	8.4	[M+H]^+^	C_28_H_30_O_17_	560.2568,463.2601,401.2549,301.1353	Calycosin-7-O-β-D-Glucoside +Hydroxylation + Glucuronidation	N, M
M15	4.74	451.1255	1.9	[M − H]^-^	C_21_H_24_O_11_	391.1213,233.1737,146.9826	Prunin + Hydrolysis	M
M16	28.19	375.1447	2.3	[M+H]^+^	C_20_H_22_O_7_	362.3213,318.2949	Pinoresinol + Hydroxylation	M
M17	36.58	361.1615	−8.5	[M+H]^+^	C_20_H_24_O_6_	318.2949,271.2694	Pinoresinol + Hydrogenation	M
M18	30.92	823.2722	6.8	[M − H]^-^	C_38_H_48_O_20_	703.5900,619.3144,543.2923,301.7546	Epimedin A + Demethylation	M
M19	30.46	1093.2760	4.4	[M − H]^-^	C_45_H_58_O_29_S	917.2354,837.2963,612.3548,588.3549, 500.2923	Epimedin A + Sulfation +Glucuronidation	M
M20	32.31	447.0942	2.0	[M − H]^-^	C_21_H_20_O_11_	409.3273,360.2741	Baicalin + Hydrogenation	N, M
M21	31.25	402.0929	−4.1	[M+H]^+^	C_20_H_17_O_9_	341.2634,239.2341,144.0968,85.0262	Baicalin + Decarboxylation	N, M
M22	31.33	431.0953	−4.5	[M+H]^+^	C_21_H_18_O_10_	400.3365,367.2788,265.2469,144.0967	Baicalin + Dehydroxylation	N, M
M23	32.65	429.0832	3.6	[M+H]^+^	C_21_H_16_O_10_	403.2796,355.0642,281.0444	Baicalin + Loss of H_2_O	M
M24	31.67	527.0458	−6.1	[M+H]^+^	C_21_H_18_O_14_S	496.3342,447.0912,358.3628,219.3563	Baicalin + Sulfation	M
M25	6.08	335.1101	−7.3	[M+H]^+^	C_17_H_18_O_7_	298.0909,170.0553,157.0571	Byakangelicol + Hydrolysis	M
M26	19.79	283.0998	7.8	[M − H]^-^	C_17_H_16_O_4_	227.1002,171.1083,157.1221	Imperatorin + Methylation	N, M
M27	7.95	301.1048	−7.4	[M+H]^+^	C_17_H_16_O_5_	254.0949,158.0916	Imperatorin +Hydroxylation + Methylation	M
M28	17.15	665.2455	0.7	[M − H]^-^	C_32_H_42_O_15_	648.2492,580.2711,478.3232,384.9600, 299.3249	Pinoresinol Diglucoside + Dehydroxylation	M
M29	17.67	699.2448	−6.7	[M+H]^+^	C_32_H_42_O_17_	616.2866,501.6044,301.1360	Pinoresinol Diglucoside + Hydroxylation	M
M30	20.44	685.2713	1.6	[M+H]^+^	C_32_H_44_O_16_	577.2784,478.2169,299.1237	Pinoresinol Diglucoside + Hydrogenation	M
M31	31.32	665.2489	7.3	[M+H]^+^	C_32_H_40_O_15_	496.3351,426.3527,400.3366	Pinoresinol Diglucoside +Loss of H_2_O	M
M32	14.88	241.1067	−6.2	[M − H]^-^	C_12_H_18_O_5_	219.1887,129.9784	Senkyunolide I + Hydrolysis	M
M33	20.14	227.1261	−7.3	[M+H]^+^	C_12_H_18_O_4_	158.0547	Senkyunolide I + Hydrogenation	N, M
M34	31.22	401.1477	8.7	[M+H]^+^	C_18_H_24_O_10_	341.2637,225.1098,158.0970	Senkyunolide I + Glucuronidation	N, M
M35	8.88	425.1403	−9.2	[M+H]^+^	C_20_H_24_O_10_	334.5176,301.1350,246.1648	Nodakenin + Hydroxylation	M
M36	27.17	411.1630	−4.8	[M+H]^+^	C_20_H_26_O_9_	387.1754,301.1340	Nodakenin + Hydrogenation	M
M37	17.27	451.1601	0.4	[M+H]^+^	C_22_H_26_O_10_	301.1376,220.2846,149.0970	Nodakenin + Acetylation	N, M
M38	23.01	399.2176	2.6	[M+H]^+^	C_24_H_30_O_5_	377.2256,301.1347,246.2371	Angelicide + Hydrolysis	M
M39	29.13	397.1987	−5.7	[M+H]^+^	C_24_H_28_O_5_	376.3007,344.2743,301.2613	Angelicide + Hydroxylation	M
M40	16.33	383.2198	−5.0	[M+H]^+^	C_24_H_30_O_4_	383.2198,301.1366,279.0891,219.1669	Angelicide + Hydrogenation	M
M41	30.55	395.2237	5.1	[M+H]^+^	C_25_H_30_O_4_	361.2603,171.1086,133.0968	Angelicide + Methylation	N, M
M42	12.48	423.2168	0.6	[M+H]^+^	C_26_H_30_O_5_	393.1228,342.2380,301.1367	Angelicide + Acetylation	M
M43	32.09	383.3541	5.6	[M+H]^+^	C_24_H_46_O_3_	311.2330,269.2165,213.1580,159.1117	Behenic Acid + Acetylation	N, M
M44	27.64	421.3016	8.1	[M+H]^+^	C_22_H_44_O_5_S	398.2364,376.2548,341.3473,301.1358	Behenic Acid + Sulfation	N, M
M45	30.63	517.3694	−8.0	[M+H]^+^	C_28_H_52_O_8_	398.3211,341.2503,238.2202	Behenic Acid + Glucuronidation	N, M
M46	17.29	451.1714	−8.3	[M+H]^+^	C_26_H_26_O_7_	301.1376,251.1149,174.0532	Yinyanghuo A +Hydroxylation + Methylation	M
M47	31.28	435.1803	0.2	[M+H]^+^	C_26_H_26_O_6_	400.3370,341.2634,239.2314,144.0968	Yinyanghuo A + Methylation	M
M48	29.14	463.1744	−1.7	[M+H]^+^	C_27_H_26_O_7_	437.1882,344.2745,285.2009	Yinyanghuo A + Acetylation	M
M49	31.56	501.1219	1.1	[M+H]^+^	C_25_H_24_O_9_S	478.3239,421.1611,184.0677	Yinyanghuo A + Sulfation	M
M50	18.37	597.1985	3.1	[M+H]^+^	C_31_H_32_O_12_	575.2120,553.2278,483.8494,421.1389	Yinyanghuo A + Glucuronidation	M
M51	34.32	459.3874	6.7	[M − H]^-^	C_30_H_52_O_3_	410.2588,358.2601,281.2685,255.2540	Lycoclavanol + Hydrogenation	N
M52	31.51	499.3775	−3.5	[M − H]^-^	C_32_H_52_O_4_	452.9407,386.9872,331.0023,171.1264	Lycoclavanol + Acetylation	N, M
M53	31.93	429.3722	−1.2	[M+H]^+^	C_29_H_48_O_2_	369.2956,267.2631,144.0972	Lycoclavanol + Loss of CH_2_O	M
M54	32.13	443.3852	−7.1	[M+H]^+^	C_30_H_50_O_2_	390.2723,310.3058,198.0487	Lycoclavanol + Dehydroxylation	N, M
M55	31.68	441.3712	−3.4	[M+H]^+^	C_30_H_48_O_2_	341.2900,266.2557,170.0526	Lycoclavanol + Loss of H_2_O	N, M
M56	32.27	539.3432	5.8	[M+H]^+^	C_30_H_50_O_6_S	510.3503,459.3975,426.3523,324.2837	Lycoclavanol + Sulfation	M
M57	13.23	715.3720	−0.2	[M+H]^+^	C_36_H_58_O_12_S	657.6741,539.3412,459.3796,301.1339	Lycoclavanol + Sulfation +Glucuronidation	M
M58	22.44	401.1209	−8.1	[M − H]^-^	C_21_H_22_O_8_	282.4231,248.9820,219.1903	Pteryxin + Hydroxylation	M
M59	31.24	405.1570	6.4	[M+H]^+^	C_21_H_24_O_8_	341.2637,239.2312,144.0970	Pteryxin + Hydrolysis	M
M60	30.35	417.1549	1.1	[M+H]^+^	C_22_H_24_O_8_	372.3060,313.2322,211.2001	Pteryxin +Hydroxylation + Methylation	N, M
M61	14.18	813.4561	−10.0	[M − H]^-^	C_42_H_70_O_15_	656.8436,522.9354,428.9405	Astragaloside A +Hydroxylation + Methylation	M
M62	21.56	801.4594	−4.6	[M+H]^+^	C_41_H_68_O_15_	703.5705,654.3243,428.1052,301.1354	Astragaloside A + Hydroxylation	M
M63	20.40	865.4169	−9.4	[M+H]^+^	C_41_H_68_O_17_S	785.5629,703.5716,638.3300,301.1352	Astragaloside A + Sulfation	M
M64	20.08	961.4925	−8.1	[M+H]^+^	C_47_H_76_O_20_	785.5650,703.5716,428.7545	Astragaloside A + Glucuronidation	M
M65	30.58	455.2672	4.6	[M − H]^-^	C_24_H_40_O_8_	327.6205,279.2313,229.4169,153.0959	Linoleic Acid + Glucuronidation	N, M
M66	34.33	297.2434	3.2	[M+H]^+^	C_18_H_32_O_3_	256.4809,219.1713	Linoleic Acid + Hydroxylation	N, M
M67	35.47	283.2618	−5.0	[M+H]^+^	C_18_H_34_O_2_	219.1727	Linoleic Acid + Hydrogenation	N
M68	16.16	241.0473	−9.0	[M+H]^+^	C_14_H_8_O_4_	219.1686,166.9098	2-Hydroxyanthraquinone +Hydroxylation	N, M
M69	24.20	401.0871	0.9	[M+H]^+^	C_20_H_16_O_9_	353.2229,242.9798,225.0612	2-Hydroxyanthraquinone +Glucuronidation	N, M
M70	36.20	267.0675	8.6	[M+H]^+^	C_16_H_10_O_4_	135.0925,113.0170	2-Hydroxyanthraquinone +Acetylation	N, M
M71	32.65	257.2473	−4.9	[M − H]^-^	C_16_H_34_O_2_	231.0028,223.0468	N-Hexadecanoic Acid +Hydrogenation	N, M
M72	29.82	447.2583	−3.8	[M − H]^-^	C_22_H_40_O_9_	391.3072,379.2647,271.2356	N-Hexadecanoic Acid +Hydroxylation + Glucuronidation	N, M
M73	33.30	285.2413	−7.9	[M − H]^-^	C_17_H_34_O_3_	271.2461,254.9988,150.3206	N-Hexadecanoic Acid +Hydroxylation + Methylation	M
M74	31.97	299.2579	−0.7	[M+H]^+^	C_18_H_34_O_3_	267.2628,144.0976	N-Hexadecanoic Acid +Acetylation	M
M75	31.38	433.2753	−9.8	[M+H]^+^	C_22_H_40_O_8_	400.3378,367.2794,257.2432	N-Hexadecanoic Acid +Glucuronidation	N, M
M76	33.02	283.2623	−6.9	[M − H]^-^	C_18_H_36_O_2_	229.4137,161.2475,135.1936	Oleic Acid + Hydrogenation	N, M
M77	28.04	311.2600	2.8	[M − H]^-^	C_19_H_36_O_3_	281.0015,219.1945,181.9141	Oleic Acid +Hydroxylation + Methylation	N, M
M78	30.81	295.2665	7.5	[M − H]^-^	C_19_H_36_O_2_	241.2525,153.0970	Oleic Acid + Methylation	N, M
M79	33.97	363.2172	−7.7	[M+H]^+^	C_18_H_34_O_5_S	283.3108,219.1717,153.0814	Oleic Acid + Sulfation	N
M80	31.97	299.2579	−0.7	[M+H]^+^	C_18_H_34_O_3_	267.2628,144.0976,85.0263	Oleic Acid + Hydroxylation	N, M
M81	32.76	459.2987	7.6	[M+H]^+^	C_24_H_42_O_8_	379.2782,283.2539	Oleic Acid + Glucuronidation	N,M
M82	29.05	313.2767	5.9	[M − H]^-^	C_19_H_38_O_3_	293.0067,231.0029,194.0952	Octadecanoic Acid +Hydroxylation + Methylation	N, M
M83	31.18	297.2789	−3.5	[M − H]^-^	C_19_H_38_O_2_	255.4978,242.3227	Octadecanoic Acid + Methylation	N, M
M84	29.78	459.2933	−6.7	[M − H]^-^	C_24_H_44_O_8_	391.7869,355.4222,283.2637	Octadecanoic Acid +Glucuronidation	N, M
M85	26.58	475.2889	−5.0	[M − H]^-^	C_24_H_44_O_9_	407.3003,351.0117,299.2498	Octadecanoic Acid +Hydroxylation + Glucuronidation	N
M86	26.71	389.1204	−6.8	[M+H]^+^	C_20_H_20_O_8_	367.1444,301.1373,242.0955	Cartilaginomarginadin +Demethylation	N, M
M87	31.24	405.1560	3.9	[M+H]^+^	C_21_H_24_O_8_	341.2637,239.2312,144.0970	Cartilaginomarginadin +Hydrogenation	M
M88	30.39	375.1092	4.7	[M+H]^+^	C_19_H_18_O_8_	313.2326,211.2001,144.0969	Cartilaginomarginadin +Demethylation + Demethylation	N, M
M89	30.35	417.1549	1.1	[M+H]^+^	C_22_H_24_O_8_	372.3060,313.2322,211.2001,144.0967, 85.0263	Cartilaginomarginadin +Methylation	N, M
M90	20.13	271.0983	6.6	[M+H]^+^	C_16_H_14_O_4_	227.1340,157.0554	Pabulenol + Demethylation	M
M91	20.96	299.1263	−5.1	[M+H]^+^	C_18_H_18_O_4_	194.0785,105.0412,77.0370	Pabulenol + Methylation	M
M92	30.40	375.1769	−8.9	[M+H]^+^	C_21_H_26_O_6_	211.1997,144.0968	Lariciresinol + Methylation	M
M93	30.55	553.1921	0.9	[M+H]^+^	C_26_H_32_O_13_	520.3345,398.3213,377.1534	Lariciresinol + Hydroxylation +Glucuronidation	N, M

Note: t_R_: retention time.

N and M represented normal and model, respectively.

#### 3.3.1 Metabolites of flavonoids

**Fig. 5:**
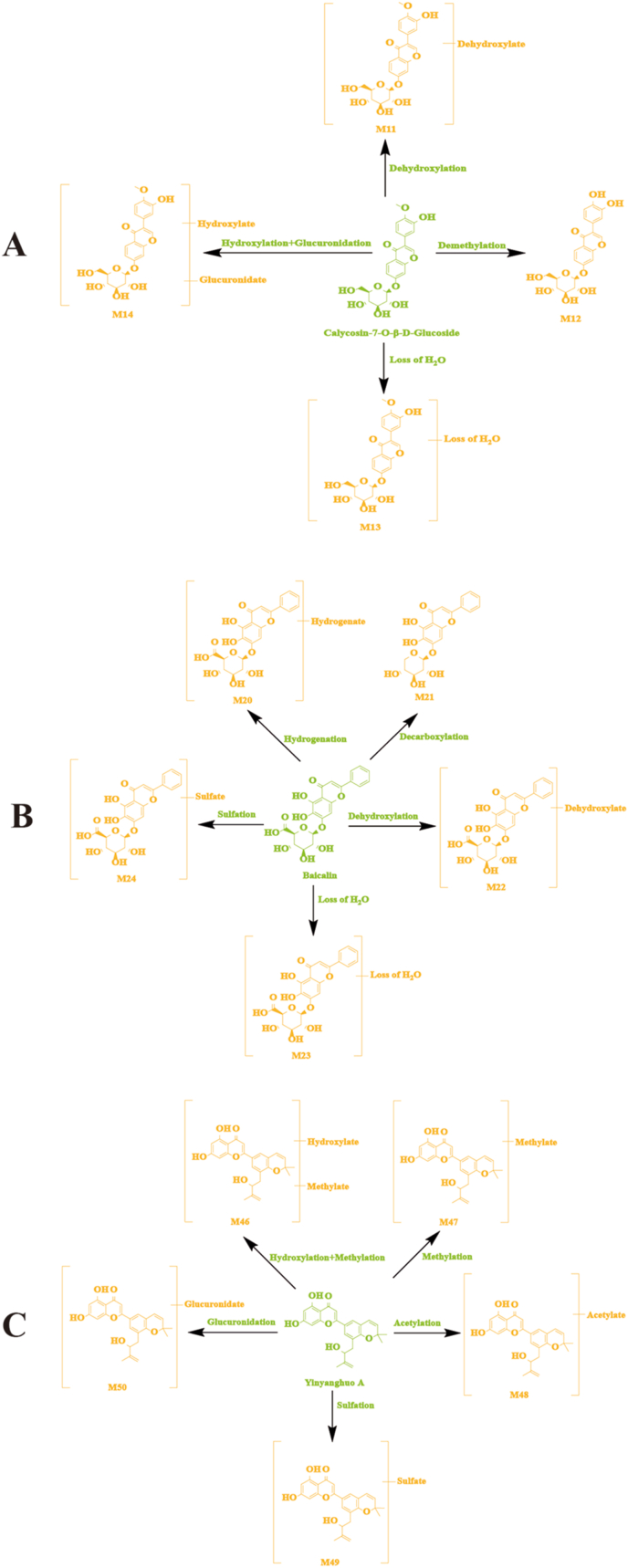
The possible metabolic pathways of calycosin-7-O-β-D-glucoside (A), baicalin (B), and yinyanghuo A (C) in normal and MCAO model rat brain.

Brain samples from normal and MCAO model rats contained 17 flavonoid-related metabolites. A representative example of calycosin-7-O-β-D-glucoside and its metabolites were used in Fig. 5A. A variety of metabolic reactions have been observed of calycosin-7-O-β-D-glucoside in rat brain samples, including dehydroxylation, demethylation, loss of H_2_O, hydroxylation, and glucuronidation. M11 was 16 Da less than that of calycosin-7-O-β-D-glucoside (A9), which meant that it might be generated by dehydroxylation from calycosin-7-O-β-D-glucoside. The fragment ions at *m/z* 301.1215 and *m/z* 246.1141 were similar to that of calycosin-7-O-β-D-glucoside. Based on the above, M11 was considered the dehydroxylated product of calycosin-7-O-β-D-glucoside. M12 was 14 Da less than that of calycosin-7-O-β-D-glucoside, which implied that M12 might be the demethylated product of calycosin-7-O-β-D-glucoside. The fragment ions at *m/z* 301.0387 and *m/z* 246.5130 were similar to that of calycosin-7-O-β-D-glucoside. According to these fragment ions, M12 was deduced as the demethylated product of calycosin-7-O-β-D-glucoside. M13 because the loss of H_2_O was 18 Da less than that of calycosin-7-O-β-D-glucoside. The fragment ions at *m/z* 301.1359 and *m/z* 246.0766 were similar to that of calycosin-7-O-β-D-glucoside. Based on the above, M13 was the product of the calycosin-7-O-β-D-glucoside losing H_2_O. M14 was 192 Da more than calycosin-7-O-β-D-glucoside, so it is speculated that the hydroxylation and glucuronidation of calycosin-7-O-β-D-glucoside formed M14. The fragment ions at *m/z* 401.2549 and *m/z* 301.1353 were the same as that of calycosin-7-O-β-D-glucoside. According to these fragment ions, M14 was considered a hydroxylated and glucuronidated product of calycosin-7-O-β-D-glucoside.

The possible metabolic pathways of calycosin-7-O-β-D-glucoside, baicalin, and yinyanghuo A in normal and MCAO model rat brain samples are shown in Fig. 5A–Fig. 5 B, and Fig. 5C. Likewise, 13 other flavonoid-related metabolites were tentatively characterized by comparing with typical neutral loss, MS fragment pattern and related studies.

#### 3.3.2 Metabolites of coumarins

**Fig. 6:**
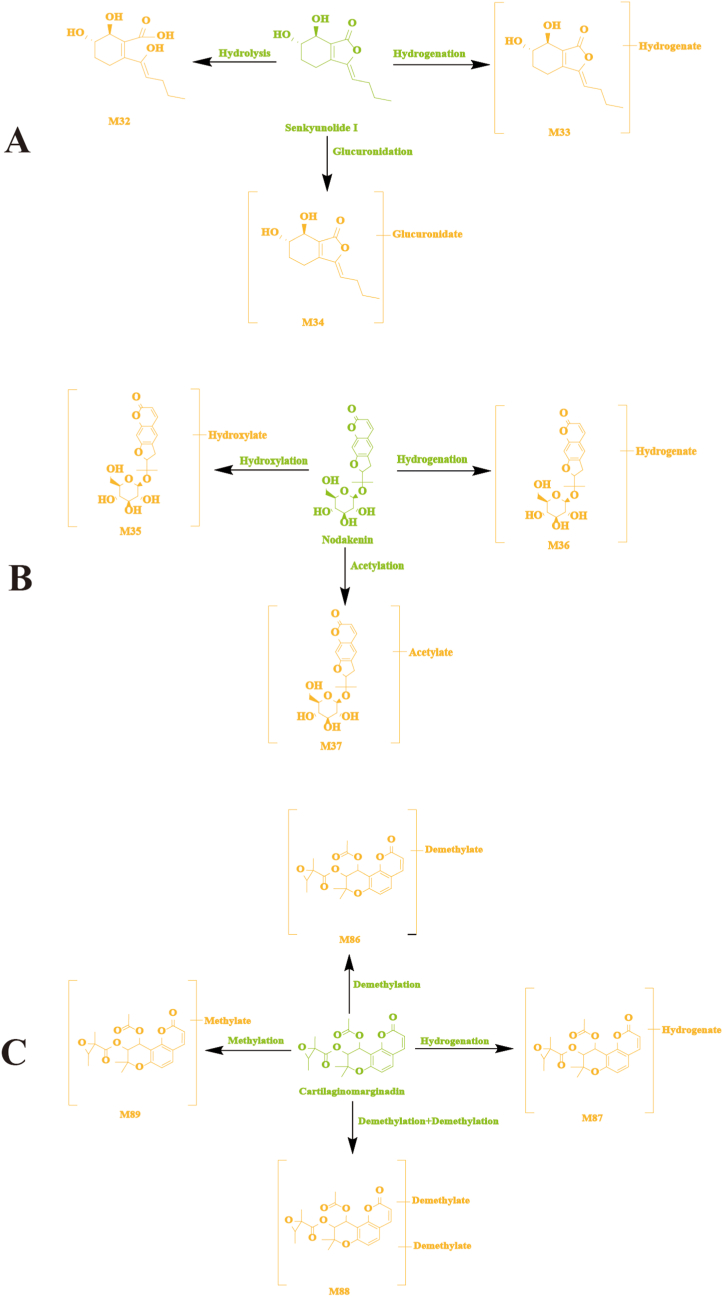
The possible metabolic pathways of senkyunolide I (A), nodakenin (B), and cartilaginomarginadin (C) in normal and MCAO model rat brain.

Coumarins generally undergo phase I reactions, as shown in Fig. 6. Brain samples from normal and MCAO model rats contained 18 coumarin-related metabolites. A representative example of senkyunolide I and its metabolites was used in Fig. 6A. Various metabolic reactions have been observed in senkyunolide I in rat brain samples, including hydrolysis, hydrogenation, and glucuronidation. M32 was 18 Da more than that of senkyunolide I (A19), suggesting it was the product of the hydrolysis reaction of senkyunolide I. The fragment ions at *m/z* 225.1098 was the same as that of senkyunolide I. Based on the above, M32 was considered the hydrolysis product of senkyunolide I. M33 was 2 Da more than that of senkyunolide I, which might have been generated by hydrogenation from senkyunolide I. The fragment ions at *m/z* 158.0547 was the same as that of senkyunolide I. Based on the above, M33 was deduced to be the hydrogenated product of senkyunolide I. M34 was 176 Da more than that of senkyunolide I, so it is speculated that M34 was formed by the glucuronidation of senkyunolide I. The fragment ions at *m/z* 225.1098 and *m/z* 158.0970 were the same as that of senkyunolide I. According to these fragment ions, M34 has been deduced as the glucuronidated product of senkyunolide I.

The possible metabolic pathways of senkyunolide I, nodakenin, and cartilaginomarginadin in normal and MCAO model rat brain samples are shown in Fig. 6A– B, and Fig. 6C. Likewise, 15 other coumarins-related metabolites were tentatively characterized by comparing with typical neutral loss, MS fragment pattern and related studies.

#### 3.3.3 Metabolites of terpenoids

**Fig. 7:**
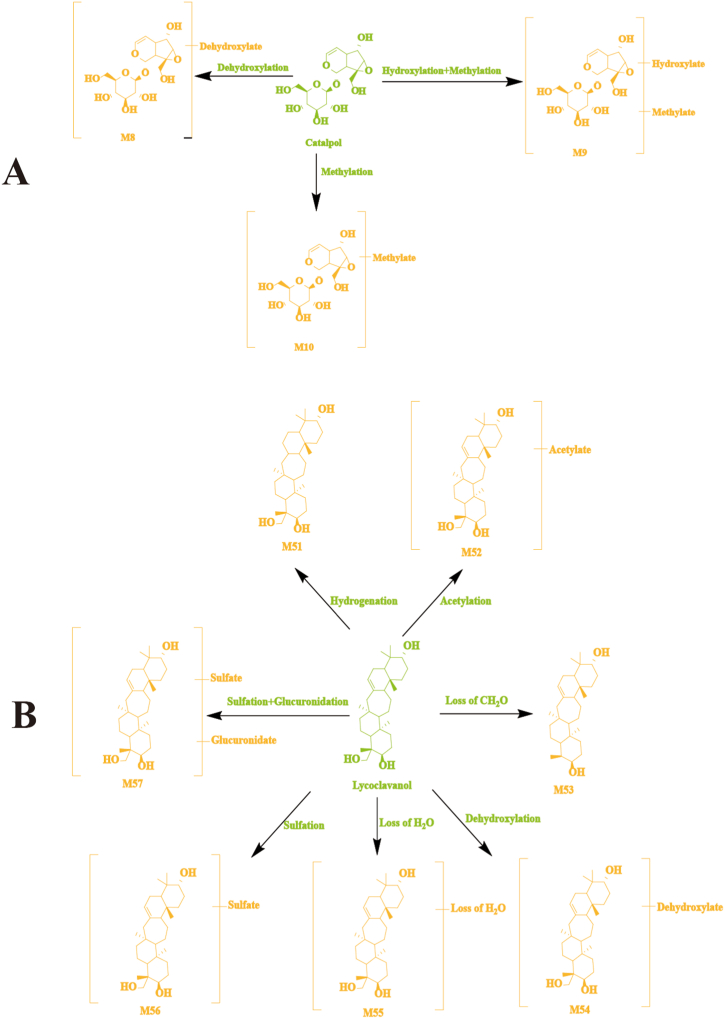
The possible metabolic pathways of catalpol (A) and lycoclavanol (B) in normal and MCAO model rat brain.

Brain samples from normal and MCAO model rats contained 10 terpenoid-related metabolites. A representative example of catalpol and its metabolites was used in Fig. 7A. Various metabolic reactions, including dihydroxylation, hydroxylation, and methylation, have been observed in catalpol in rat brain samples. M8 was 16 Da less than that of catalpol (A8), which meant that it might be generated by dehydroxylation from catalpol. The fragment ions at *m/z* 219.5524 was similar to that of catalpol. Based on the above, M8 was considered the dehydroxylated product of catalpol. M9 was 30 Da greater than that for catalpol, which implied that M9 might be the hydroxylation and methylation product of catalpol. The fragment ions at *m/z* 357.6841 and *m/z* 301.1197 were the same as that of catalpol. According to these fragment ions, M9 was deduced as the hydroxylated and methylated product of catalpol. M10 was 14 Da more than catalpol, so it is speculated that the methylation of catalpol formed M10. The fragment ions at *m/z* 357.1352, *m/z* 301.1369, and *m/z* 219.1721 were the same as that of catalpol. According to these fragment ions, M10 was deduced as the methylated product of catalpol.

The possible metabolic pathways of catalpol and lycoclavanol in normal and MCAO model rat brain samples are shown in Fig. 7A and B, respectively. Likewise, seven other terpenoid-related metabolites were tentatively characterized by comparing with typical neutral loss, MS fragment pattern, and related studies.

#### 3.3.4 Identification of other related metabolites

**Fig. 8:**
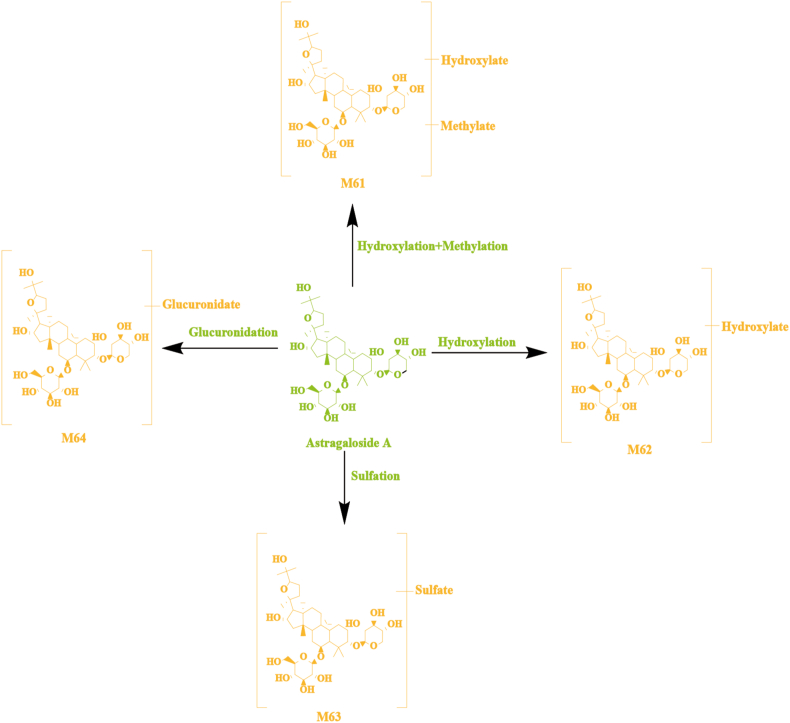
The possible metabolic pathways of astragaloside A in normal and MCAO model rat brain.

Brain samples from normal and MCAO model rats contained 48 other related metabolites. A representative example of astragaloside A and its metabolites was used in Fig. 8A. Various metabolic reactions have been observed of astragaloside A in rat brain samples, including hydroxylation, methylation, sulfation, and glucuronidation. M61 was 30 Da more than that of astragaloside A (A30), suggesting it was the product of astragaloside A's hydroxylation and methylation reaction. The fragment ions at *m/z* 522.9354 and *m/z* 428.9405 were the same as that of astragaloside A. Based on the above, M61 was deduced as the hydroxylated and methylated product of astragaloside A. M62 was 16 Da greater than that for astragaloside A, so it is speculated that M62 was formed by the hydroxylated of astragaloside A. The fragment ions at *m/z* 703.5705 and *m/z* 428.1052 were the same as that of astragaloside A. Based on the above, M62 was deduced as the hydroxylated product of astragaloside A. M63 was 80 Da greater than that for astragaloside A, which implied M63 might be the sulfation product of astragaloside A. The fragment ions at *m/z* 703.5716 was the same as that of astragaloside A. According to these fragment ions, M63 was deduced as the sulfated product of astragaloside A. M64 was 176 Da greater than that for astragaloside A, suggesting it was the product of the glucuronidation reaction of astragaloside A. The fragment ions at *m/z* 703.5716 and *m/z* 428.7545 were the same as that of astragaloside A. According to these fragment ions, M64 was deduced as the glucuronidated product of astragaloside A.

**Fig. 9:**
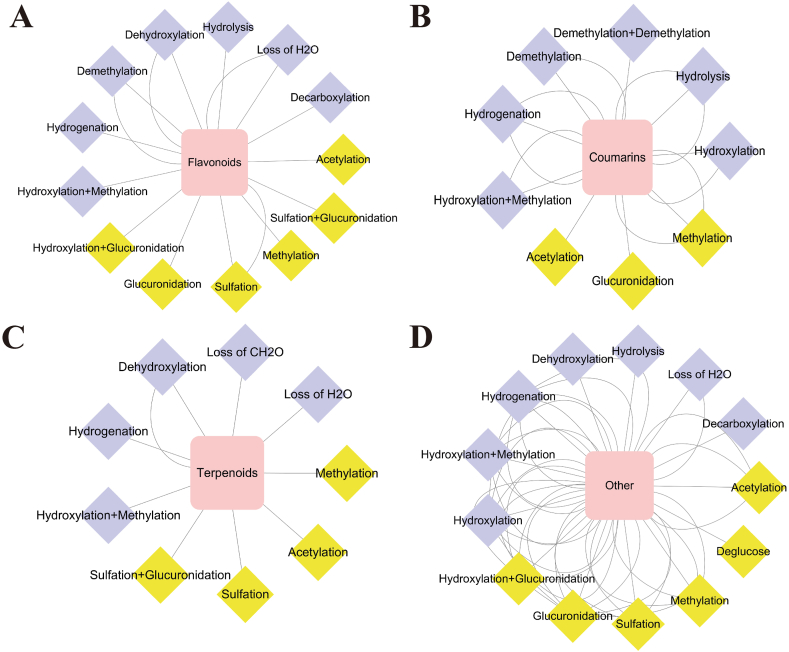
The possible metabolic pathways of four main type constituents in DZP in normal and MCAO model rat brain: flavonoids (A), coumarins (B), terpenoids (C), and other (D) related metabolites; phase I metabolic routes(purple); phase II metabolic routes(yellow).

The possible metabolic pathways of astragaloside A in normal and MCAO model rat brain samples are shown in Fig. 8. Likewise, 44 additional metabolites, including vanillic acid metabolites M1, M2, gallic acid metabolites M4—M7, pinoresinol metabolites M16, M17, pinoresinol diglucoside metabolites M28—M31, angelicide metabolites M38—M42 were also tentatively characterized by comparing with typical neutral loss, MS fragment pattern and related studies. Detailed information is provided in Table 2.

### 3.4 The pathway analysis of phase I metabolites

#### 3.4.1 Decarboxylation

The brain samples from rats with normal and MCAO models contained two decarboxylated (M4, M21) related metabolites.M4 was an example. M4 was 45 Da less than that of gallic acid (A3), meaning it might be generated by decarboxylation from gallic acid. The fragment ions at *m/z* 124.0219 and *m/z* 78.9714 were similar to gallic acid. Based on the above, M4 was deduced as the decarboxylated product of gallic acid.

#### 3.4.2 Dehydration

The brain samples from rats with normal and MCAO models contained five dehydrated (M1, M13, M23, M31, M55) related metabolites. M1 was taken as an example. M1 because the loss of H_2_O was 18 Da less than that of vanillic acid (A1). The fragment ions at *m/z* 124.9213 and *m/z* 78.3732 were the same as that of vanillic acid. According to these fragment ions, M1 was deduced to be the product that vanillic acid lost H_2_O.

#### 3.4.3 Demethylation

The brain samples from rats with normal and MCAO models contained four demethylated (M12, M18, M86, M90) related metabolites. M18 was taken as an example. M18 was 14 Da smaller than that for epimedin A (A11), which implied that M18 might be the demethylation product of epimedin A. The fragment ions at *m/z* 703.5900, *m/z* 543.2923, and *m/z* 301.7546 were similar to that of epimedin A. According to these fragment ions, M18 was deduced as the demethylated product of epimedin A.

#### 3.4.4 Hydrogenation

The brain samples from rats with normal and MCAO models contained 11 hydrogenated (M17, M20, M30, M33, M36, M40, M51, M67, M71, M76, M87) related metabolites. M17 was taken as an example. M17 was 2 Da greater than that for pinoresinol (A10), suggesting it was the product of the hydrogenation reaction of pinoresinol. The fragment ions at *m/z* 271.2694 was the same as that of pinoresinol. Based on the above, M17 was considered the hydrogenated product of pinoresinol.

#### 3.4.5 Dehydroxylation

The brain samples from normal and MCAO models of rats contained five dehydroxylated (M8, M11, M22, M28, M54) related metabolites. M28 was taken as an example. M28 was 16 Da smaller than that for pinoresinol diglucoside (A16), which implied that M28 might be the dehydroxylation product of pinoresinol diglucoside. The fragment ions at *m/z* 478.3232 and *m/z* 299.3249 were similar to that of pinoresinol diglucoside. According to these fragment ions, M28 was deduced as the dehydroxylated product of pinoresinol diglucoside.

#### 3.4.6 Hydroxylation

The brain samples from rats with normal and MCAO models contained nine hydroxylated (M16, M29, M35, M39, M58, M62, M66, M68, M80) related metabolites. M39 was taken as an example. M39 was 16 Da greater than that for angelicide (A20), suggesting it was the product of the hydroxylation reaction of angelicide. The fragment ions at *m/z* 376.3007 and *m/z* 301.2613 were the same as that of angelicide. According to these fragment ions, M39 was deduced as the hydroxylated product of angelicide.

### 3.5 The pathway analysis of phase II metabolites

#### 3.5.1 Methylation

The brain samples from rats with normal and MCAO models contained nine methylated (M10, M26, M41, M47, M78, M83, M89, M91, M92) related metabolites. M26 was taken as an example. M26 was 14 Da greater than that for imperatorin (A15), so it is speculated that the methylation of imperatorin formed M26. The fragment ions at *m/z* 227.1002 and *m/z* 157.1221 were the same as that of imperatorin. According to these fragment ions, M26 was deduced as the methylated product of imperatorin.

#### 3.5.2 Acetylation

The brain samples from rats with normal and MCAO models contained seven acetylated (M37, M42, M43, M48, M52, M70, M74) related metabolites. M52 was taken as an example. M52 was 42 Da greater than that for lycoclavanol (A25), which implied that M52 might be the acetylation product of lycoclavanol. The fragment ions at *m/z* 331.0023 and *m/z* 171.1264 were the same as that of lycoclavanol. According to these fragment ions, M52 was deduced as the acetylated product of lycoclavanol.

#### 3.5.3 Sulfation

The brain samples from rats with normal and MCAO models contained seven sulfated (M5, M24, M44, M49, M56, M63, M79) related metabolites. M24 was taken as an example. M24 was 80 Da greater than that for baicalin (A13), suggesting it was the product of the sulfation reaction of baicalin. The fragment ions at *m/z* 219.3563 was the same as that of baicalin. Based on the above, M24 was considered to be the sulfated product of baicalin.

#### 3.5.4 Glucuronidation

The brain samples from rats with normal and MCAO models contained 10 glucuronidated (M7, M34, M45, M50, M64, M65, M69, M75, M81, M84) related metabolites. M7 was taken as an example. M7 was 176 Da greater than that for gallic acid (A3), which implied that M7 might be the glucuronidation product of gallic acid. The fragment ions at *m/z* 171.0298 was the same as gallic acid. Based on the above, M7 was deduced to be the glucuronidated product of gallic acid.

### 3.6 Comparison of metabolites in brain samples between normal and MCAO rats treated by DZP

The types and numbers of the metabolites in the brain samples were different between MCAO rats and normal rats. As shown in Table 2, normal and MCAO model rat brain samples contained 47 and 86 metabolite components, respectively. There were 40 metabolites both in normal and MCAO model rat brains. Seven metabolites (M1, M2, M7, M51, M67, M79, and M85) were only detected in normal rat brain samples, while 46 metabolites were found only in MCAO model rat brain samples.

**Fig. 10:**
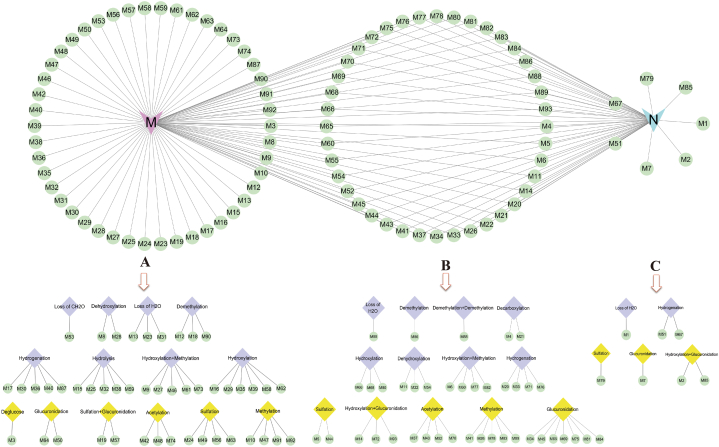
Comparison of metabolites and metabolic pathways in normal and MCAO model rat brain samples: the metabolites discovered only in MCAO rats (A), the metabolites discovered in both MCAO and normal rats (B), the metabolites discovered only in normal rats (C).

Glucuronidation, methylation, acetylation, and sulfation are phase II metabolic routes of DZP, while hydrogenation, hydroxylation, and dehydroxylation are phase I metabolic routes. Based on the number of metabolites in these metabolic pathways, hydrogenation, glucuronidation, hydroxylation, and methylation were the main metabolic pathways. Furthermore, the metabolic routes in MCAO rat brain samples were more diverse than those in normal rat brain samples (Fig. 10).

As the oral dosage, the metabolism of DZP was greatly affected by the intestinal flora. The microbiota-gut-brain axis has recently been found to communicate bidirectionally between the brain and gut, which provides novel avenues for both the prevention and treatment of stroke [21,22]. In the pathological condition of cerebral ischemia, the intestinal flora will change significantly, affecting the metabolism of drugs in the intestine and the activities of metabolic enzymes in the liver. Therefore, the metabolic differences between normal and MCAO rats in this study would provide a valuable reference for the further search for effective substances of DZP associated with the treatment of cerebral ischemia.

## 4 Conclusion

UHPLC-Q-TOF-MS/MS was used to detect and identify metabolites in rat brain samples of normal and MCAO model rats administered with DZP. Our analytical strategy led to a relatively comprehensive identification of DZP metabolites. It was tentatively found that 43 prototypes and 93 metabolites could be identified in rat brain samples. Normal and MCAO model rat brain samples contained 19 prototype components. Eight prototype components were only detected in normal rat brain samples, while 16 were found only in MCAO model rat brain samples. A marked difference was observed in comparing prototype components in brain samples from normal and MCAO rats. 47 metabolites were identified in healthy rats, while 86 were detected in MCAO model rats. Both normal and MCAO model rat brain samples contained 40 metabolites. Seven metabolites were only detected in normal rat brain samples, while 46 were found only in MCAO model rat brain samples. The comparison of metabolites in brain samples of normal and MCAO rats differed markedly.

Glucuronidation, methylation, acetylation, and sulfation are phase II metabolic routes of DZP, while hydrogenation, hydroxylation, and dehydroxylation are phase I metabolic routes. Based on the number of metabolites in these metabolic pathways, hydrogenation, glucuronidation, hydroxylation, and methylation were the main metabolic pathways.

The types of prototype components in the brain are significantly smaller than those in plasma, urine, and feces, and the number of prototype components in the brain is also smaller than in the periphery. We previously studied the metabolic pathways of DZP in rat plasma, urine, and feces; the compounds of DZP undergo phase I metabolic routes of hydroxylation, dehydroxylation, and hydrogenation and phase II metabolic routes of acetylation, sulfation, glucuronidation, and methylation. The metabolic pathways of DZP in rat plasma, urine, and feces are the same as those of DZP in the brain. The primary metabolites of DZP in rat plasma, urine, and feces include flavonoids, phenolic acids, terpenoids, and alkaloids, while in the brain, the metabolites mainly include flavonoids, coumarins, and terpenoids. The types and the number of metabolites identified in the brain are less than those in plasma, urine, and feces. The possible reason is that the metabolites mainly come from peripheral metabolism, and only parts of them can enter the brain through the blood-brain barrier.

For the critical prototypic and metabolic components in brain tissue, further quantitative analysis with triple quadrupole is needed. In addition, high-resolution mass spectrometry can only identify the components in the brain, and their spatial distribution can be further analyzed by mass spectrometry imaging. In all, this study provides a valuable reference for further research into effective substances for treating cerebral ischemia.

## Funding statement

Innovative Project in Science and Technology of China Academy of Chinese Medical Sciences (CI2021B017-07， CI2021A05208), Key Research and Development Project of Shandong Province of China (No. 2021SFGC1202). 10.13039/100014717National Natural Science Foundation of China (No. 81973711), Autonomic Project of China Academy of Chinese Medical Sciences (No.ZXKT22017, ZZ13-019)**.**

## Data availability statement

Data included in article/supplementary material/referenced in article.

## Additional information

No additional information is available for this paper.

## CRediT authorship contribution statement

**Huaqing Lai:** Writing – original draft. **Guanghuan Tian:** Investigation, Formal analysis. **Fuzhu Pan:** Investigation, Formal analysis. **Jianyong Zhang:** Conceptualization. **Hongwei Wu:** Conceptualization.

## Declaration of competing interest

The authors declare that they have no known competing financial interests or personal relationships that could have appeared to influence the work reported in this paper.
